# Effect of permeability and fractures on oil mobilization of unconventional resources during CO_2_ EOR using nuclear magnetic resonance

**DOI:** 10.1038/s41598-021-92158-3

**Published:** 2021-06-16

**Authors:** Huanquan Sun, Haitao Wang, Zengmin Lun

**Affiliations:** 1 Sinopec, Beijing, 100728 China; 2 Petroleum Exploration and Production Research Institute (PEPRIS), Sinopec, Beijing, 100083 China

**Keywords:** Carbon capture and storage, Fossil fuels

## Abstract

CO_2_ EOR (enhanced oil recovery) will be one of main technologies of enhanced unconventional resources recovery. Understanding effect of permeability and fractures on the oil mobilization of unconventional resources, i.e. tight oil, is crucial during CO_2_ EOR process. Exposure experiments based on nuclear magnetic resonance (NMR) were used to study the interaction between CO_2_ and tight oil reservoirs in Chang 8 layer of Ordos Basin at 40 °C and 12 MPa. Effect of permeability and fractures on oil mobilization of exposure experiments were investigated for the different exposure time. The oil was mobilized from matrix to the surface of matrix and the oil recovery increased as the exposure time increased. The final oil recovery increased as the core permeability increased in these exposure experiments. Exposure area increased to 1.75 times by fractures resulting in that oil was mobilized faster in the initial stage of exposure experiment and the final oil recovery increased to 1.19 times from 28.8 to 34.2%. This study shows the quantitative results of effect of permeability and fractures on oil mobilization of unconventional resources during CO_2_ EOR, which will support CO_2_ EOR design in Chang 8 layer of Ordos Basin.

## Introduction

Unconventional oil and gas resources such as tight oil reservoirs have become very significant resources in the world^1^. At present, the depletion development is carried out in most tight oil reservoirs after horizontal wells multi-stage fracturing process^2^. The initial production is high, but the production declines sharply^3^. So it is urgent to find ways to enhance tight oil reservoirs recovery^4^. CO_2_ EOR has been recognized as a potential way to enhance tight oil reservoirs recovery and to reduce CO_2_ emissions and greenhouse gas effects^5,6^.

The reservoirs in Chang 8 layer of Ordos Basin are tight sandstones with average matrix permeability of 0.3 mD. Horizontal well fracturing was carried out. The oil in fractures was easily produced. The initial oil production was high. After a period of time the oil in the fracture was produced in large quantities. And the oil remaining in the fractures and oil in the matrix were slowly produced. Oil production rapidly decreased and oil recovery was still very low. More importantly, all wells of Honghe Oilfield and Jinghe Oilfield of Sinopec in Ordos Basin were shut off due to the low oil price in previous years. Recently, oil well production has been planned using CO_2_ fracturing technology and CO_2_ enhanced oil recovery technology. In order to select befitting well and to optimize CO_2_ fracturing design and to determine the CO_2_ enhanced oil recovery parameters, it is necessary to understand the effect of permeability and fractures on oil mobilization of tight oil reservoirs exposed to CO_2_.

NMR T_2_ technology at high temperature and high pressure conditions has been used to study the oil mobilization during CO_2_ EOR process of tight oil reservoirs because of the advantage of fluid quantitative identification in micro-nano pores and CO_2_ silence^7,8^. Many studies have shown that the oil in tight matrix was mobilized after CO_2_ injection in tight oil reservoirs. Mechanisms of oil mobilization during CO_2_ EOR process of tight oil reservoirs have been determined, i. e. oil swelling and concentration-driven diffusion of hydrocarbons caused by the CO_2_ diffusion^9^. NMR T_2_ technology at high temperature and high pressure conditions was used to investigate the exposure process between CO_2_ and tight sandstone cores with different permeability (< 1mD) and simulated fracture (increasing contact area) in this study. Many studies showed that oil recovery increased as permeability of tight matrix increased and fractures were also beneficial for CO_2_ EOR in tight oil reservoirs^7,10,11^. In this paper exposure experiments of sandstone cores with different permeability and two types of fracture networks (simple fractures and complex fractures) were designed to investigate the effect of permeability and fracture on oil mobilization using NMR in the pore scale. This study shows the quantitative results for effect of permeability and fractures on oil mobilization of tight sandstone cores exposed to CO_2_ in Chang 8 layer of Ordos Basin. All conclusions forcefully support CO_2_ EOR design in Honghe Oilfield and Jinghe Oilfield of Sinopec.

## Results

### NMR T_2_ spectra of CO_2_ exposure experiments

**Figure 1:**
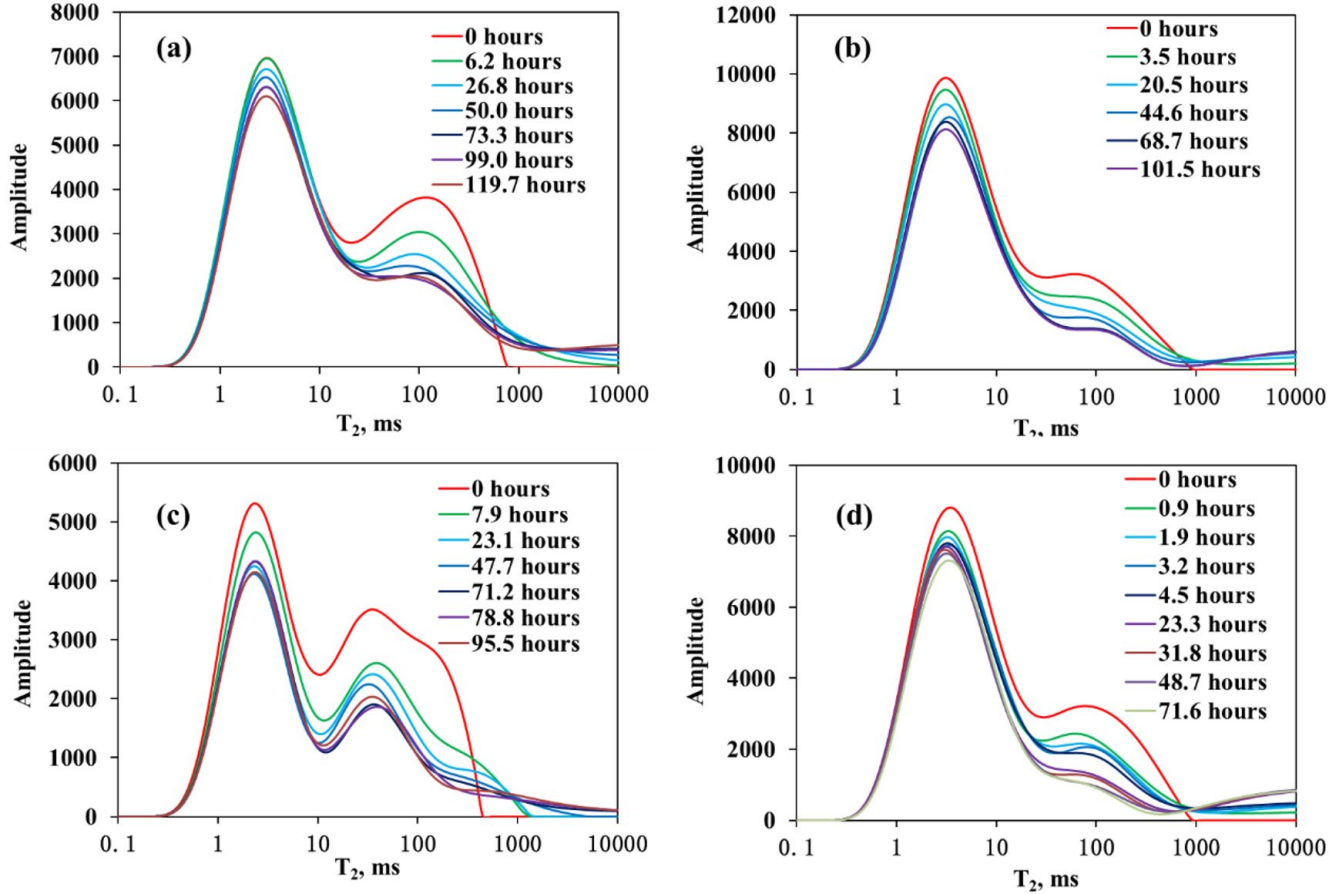
NMR T_2_ spectra of CO_2_ exposure experiments of the four cores: (**a**) 0.218 mD (sample 1#) without the fractures; (**b**) 0.598 mD (sample 2#) without the fractures; (**c**) 0.770 mD (sample 3#) without the fractures; (**d**) 0.598 mD (sample 4#) with the fractures.

The CO_2_ Exposure experiments of four cores were performed at 40 °C and 12 MPa. NMR T_2_ spectra of CO_2_ exposure experiments of the four cores: (a) 0.218 mD (sample 1#) without the fractures; (b) 0.598 mD (sample 2#) without the fractures; (c) 0.770 mD (sample 3#) without the fractures; (d) 0.598 mD (sample 4#) with the fractures are shown in Fig. 1. Many similar characteristics can be observed from these NMR T_2_ spectra.

First, NMR T_2_ spectrum amplitude gradually reduced as exposure time increased. This result shows that oil was gradually mobilized as exposure time increased. Second, oil initially existed in the matrix with a maximum relaxation time in the original saturated core. After the CO_2_ exposure experiment started, oil signal could be observed in the zone with relaxation times slower than maximum relaxation time. This phenomenon shows that oil was mobilized from matrix to the surface of matrix. For example, oil initially existed in the matrix with the maximum relaxation time of 645 ms in the original saturated core or 0 h in Fig. 1a. After the CO_2_ exposure experiment started, oil signal could be observed in the zone with relaxation times slower than maximum relaxation time of 645 ms. The zone with relaxation times slower than maximum relaxation time of 645 ms was the surface of this core. Third, reduction of NMR T_2_ spectrum amplitude in the zone of the slow relaxation time (29 ms < T_2_ ≤ the maximum relaxation time of the matrix ) was bigger than that in the zone of the fast relaxation time (T_2_ ≤ 29 ms). This result indicates that oil in big pores could be mobilized much more than that in small pores. Fourth, reduction of NMR T_2_ spectrum amplitude in the initial stage of exposure experiment was bigger than that in the late stage of exposure experiment. This result shows that oil mobilization in the initial stage of exposure experiment was faster than that in the late stage of exposure experiment, for example, the initial stage from 0 to 6.2 h in the Fig. 1a, the initial stage from 0 to 3.5 h in the Fig. 1b, the initial stage from 0 to 7.9 h in the Fig. 1c, the initial stage from 0 to 0.9 h in the Fig. 1d. For the NMR T_2_ spectra of CO_2_ exposure experiments of cores with different permeability (Fig. 1a–c), reduction of NMR T_2_ spectrum amplitude at the same CO_2_ exposure time increased as permeability increased. For the NMR T_2_ spectra of CO_2_ exposure experiments of cores with and without fractures (Fig. 1b,d), reduction rate of NMR T_2_ spectrum amplitude of the core with the fractures was bigger than that without the fractures in the initial stage of exposure experiments.

### Effect of permeability on oil recoveries in the pore scale

**Figure 2:**
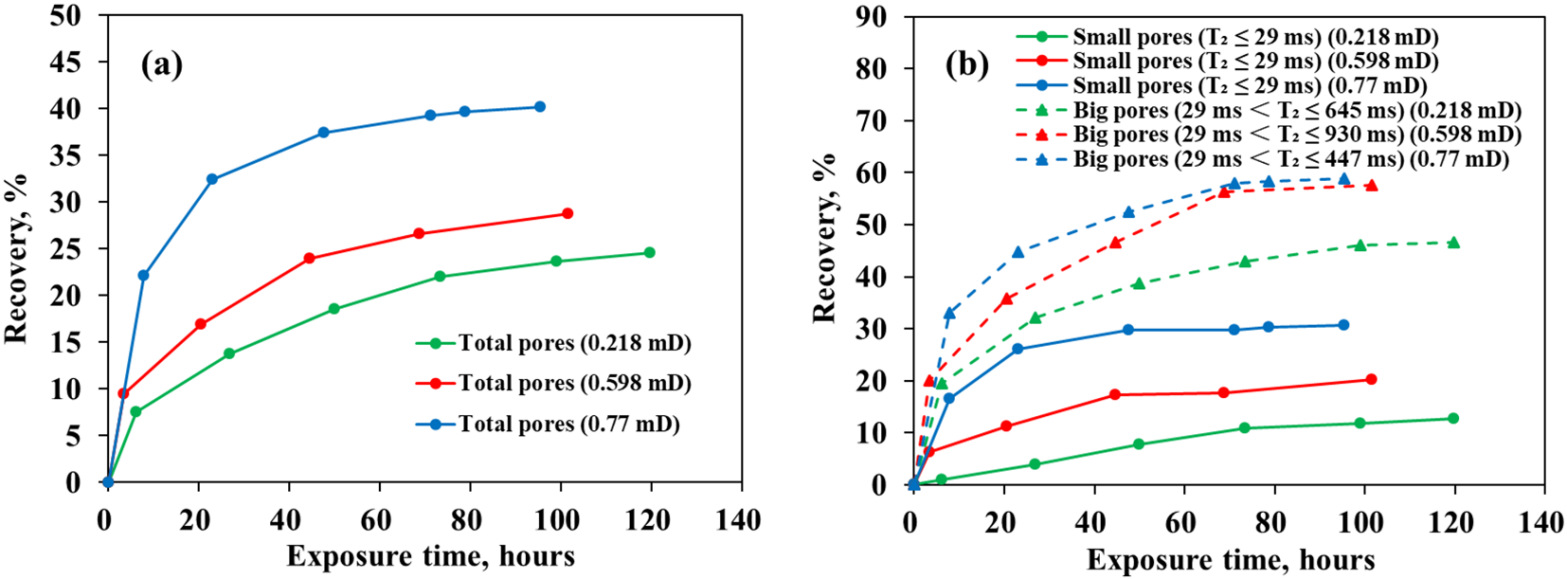
The relationships between recovery and exposure time for three cores with different permeability: (**a**) total pores; (**b**) small pores and big pores.

The relationships between oil recovery and exposure time for three cores (sample 1#, 2#, 3#) with different permeability are shown in Fig. 2. The oil recovery of CO_2_ exposure experiment was calculated by the following Eq. (1).

1 $$ R_{t}  = \frac{{A_{0}  - A_{t} }}{{A_{0} }}{\text{~}} $$

where R_t_ was the oil recovery factor at time t, A_0_ was the sum of the initial NMR signal amplitude, A_t_ was the sum of the NMR signal amplitude at time t. The small pores were defined as the fast relaxation time zone of T_2_ ≤ 29 ms. The big pores were defined as the slow relaxation time zone of 29 ms < T_2_ ≤ the maximum relaxation time of the matrix. The total pores were defined as the sum of small pores and big pores.

In Fig. 2a, it can be observed that the oil recovery of the total pores increased as the exposure time increased for the cores with the permeability of 0.218 mD, 0.598mD and 0.77mD, respectively. The oil recovery of the total pores increased as the core permeability increased during CO_2_ exposure experiment. In Fig. 2b, it is obvious that the oil recovery of big pores was higher than that of small pores for the core with same permeability. The oil recovery of big pores, small pores increased as the core permeability increased. It is worth noting that the increase of oil recovery of small pores was bigger than that of big pores as core permeability increased. That is mainly attributed to high connectivity of high permeability core. So oil in small pores of high permeability core could be contacted and mobilized more easily.

In Fig. 2a, the oil recovery sharply increased in the initial stage of the exposure experiment as exposure time increased, especially for the core with 0.770 mD. However from Fig. 2b, that phenomenon could not be obviously observed in small pores of the cores with 0.218 mD and 0.598 mD. This result shows that oil mobilization in small pores needed longer exposure time for the cores with 0.218 mD and 0.598 mD due to low connectivity. So during CO_2_ fracturing and EOR processes, reservoirs with lower permeability need more soaking time in order to mobilize oil in small pores. For CO_2_ huff-n-puff process high pressure^12^, high injection rate, fracture network and high permeability are beneficial for oil mobilization in the initial exposure stage. More soaking time is needed to mobilize the oil in small pores of matrix in the later exposure stage. The favorable fractures are to form the homogeneous fracture network with the maximum exposure area.

### Effect of the fractures on oil recoveries in the pore scale

**Figure 3:**
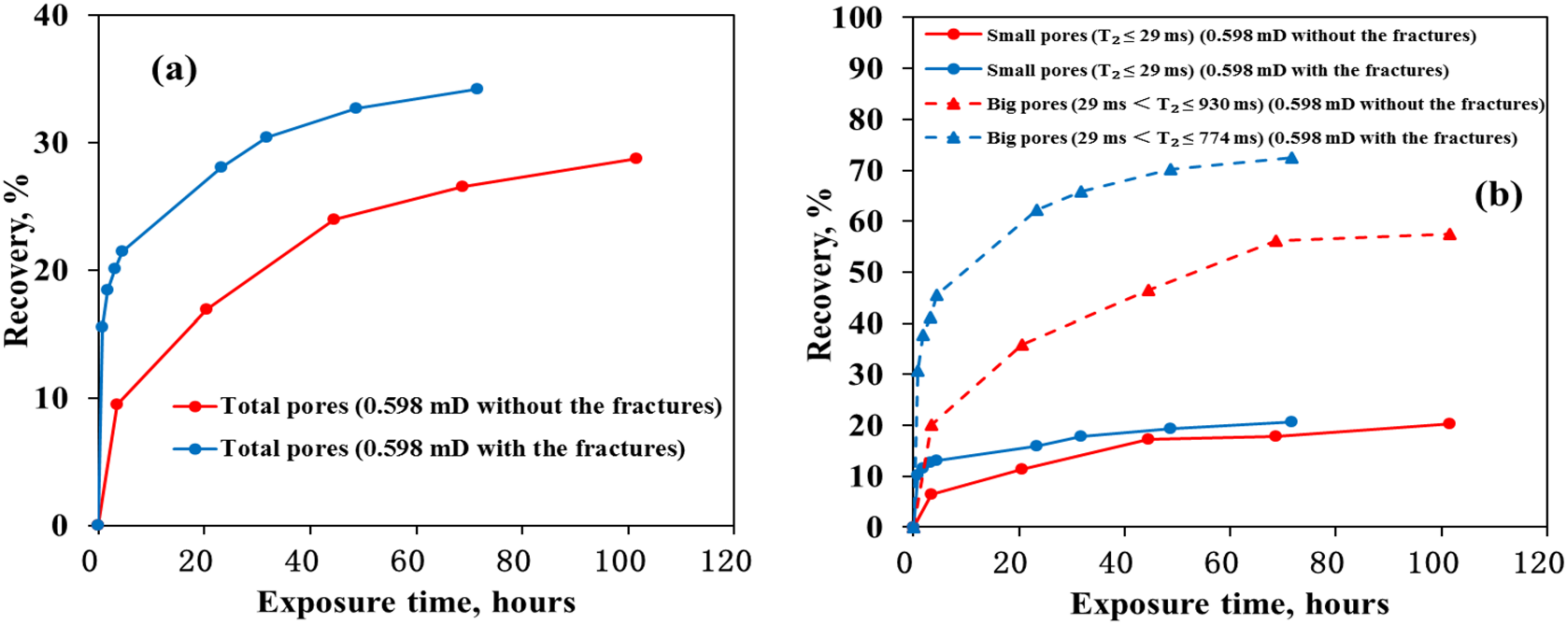
The relationships between recovery and exposure time for the cores with and without fractures: (**a**) total pores; (**b**) small pores and big pores.

The relationships between oil recovery and exposure time for the cores with and without fractures are shown in Fig. 3. The oil recovery of CO_2_ exposure experiment was also calculated by the Eq. (1). In Fig. 3a, the oil recovery of the total pores for the core with the fractures was higher than that for the core without the fractures. Especially, in the initial stage of the exposure experiment the oil recovery of the total pores for the core with the fractures increased sharply as exposure time increased. This result is mainly attributed to the increase of contact area between CO_2_ and matrix, i.e. ratio of the external surface area and volume (Table 1), generated from the fractures. Exposure area increased to 1.75 times by fractures and the final oil recovery increased to 1.19 times from 28.8% to 34.2%. The increase of the oil recovery of big pores for the core with the fractures was more significant than that for the core without the fractures (Fig. 3b). This indicates the increase of contact area generated from fractures was very beneficial for oil mobilization in the big pores. It is also obvious that the oil recovery of big pores was higher than that of small pores for the core with and without the fracture.

**Table 1:** Samples.

Samples	Porosity (%)	Pemeability (mD)	Ratio of the external surface area and volume (m^2^/m^3^)	
1#	9.5	0.218	2	Matrix
2#	12.2	0.598	2	Matrix
3#	12.5	0.770	2	Matrix
4#	11.9	0.590	3.5	Matrix with fractures

## Discussion

Exposure experiments between CO_2_ and tight sandstone cores were performed. Interaction between CO_2_ and oil and oil mobilization were studied using NMR T_2_ spectrum in this paper. There were tight sandstone cores with three permeability of 0.218 mD, 0.598 mD and 0.770 mD and with and without fractures in exposure experiments. NMR T_2_ spectrum of tight sandstone cores were recorded and analyzed in situ under different exposure time.

The characteristics of oil mobilization of exposure experiments are summarized through experiments : (1) the oil was mobilized by CO_2_ from matrix to the surface of the matrix (fractures). (2) the oil in big pores was mobilized much more than that in small pores. (3) oil mobilization and recovery increased as exposure time increased, especially in the initial exposure stage. The effect of permeability and fractures on oil mobilization was significant. The final oil recoveries of tight sandstone cores with the permeability of 0.218 mD, 0.598 mD and 0.770 mD were 24.5%, 28.8% and 40.1%, respectively and the final oil recovery increased as the core permeability increased in these exposure experiments. Moreover, as the core permeability increased the final oil recovery increased curvilinearly not linearly. This result is mainly attributed to high connectivity of high permeability core. It is very important to select befitting well according to the final oil recovery of CO_2_ EOR.

Two types of fracture networks (simple fractures and complex fractures) were designed to investigate the effect of fracture on oil mobilization in the pore scale. Exposure area was used to characterize fracture. Exposure area increased to 1.75 times by fractures, which resulted in that the final oil recovery increased to 1.19 times from 28.8% to 34.2%. This result shows that fracturing is effective for CO_2_ mobilizing oil in tight matrix. The increasing final oil recovery did not match the increasing exposure area. It need more data to illustrate this phenomenon.

The oil was mobilized faster and much more in the cores with high permeability or fractures. From the point of view of oil mobilization efficiency these zones with higher permeability and fractures are mainly sweet spot of CO_2_ injection. However, the presence of fractures and high permeability zones result the strong heterogeneity. These zones with higher permeability and fractures are the CO_2_ channeling and not beneficial for sweep efficiency. So these are very important for CO_2_ EOR to select sweet spot and to optimize the injection parameter during CO_2_ huff-n-puff or displacing process. For CO_2_ huff-n-puff process high pressure, high injection rate, fracture network and high permeability are beneficial for final recovery in the initial exposure stage. More soaking time is needed to mobilize the oil in small pores of matrix in the later exposure stage. The favorable fractures are to form the homogeneous fracture network with the maximum exposure area. For CO_2_ displacing process high pressure, suitable injection rate, suitable fracture network and suitable permeability are beneficial for final recovery with consideration of the gas channeling. The fracture parallel to the displacement direction easily result in gas channeling. And fractures at a certain angle to the displacement direction improve the sweep efficiency and oil mobilization.

This paper provides qualitative and quantitative data on the effect of exposure time, permeability and fractures on oil mobilization to support CO_2_ fracturing design optimization and CO_2_ EOR pilot test area selection in tight oil reservoirs.

## Methods

### Materials

**Figure 4:**
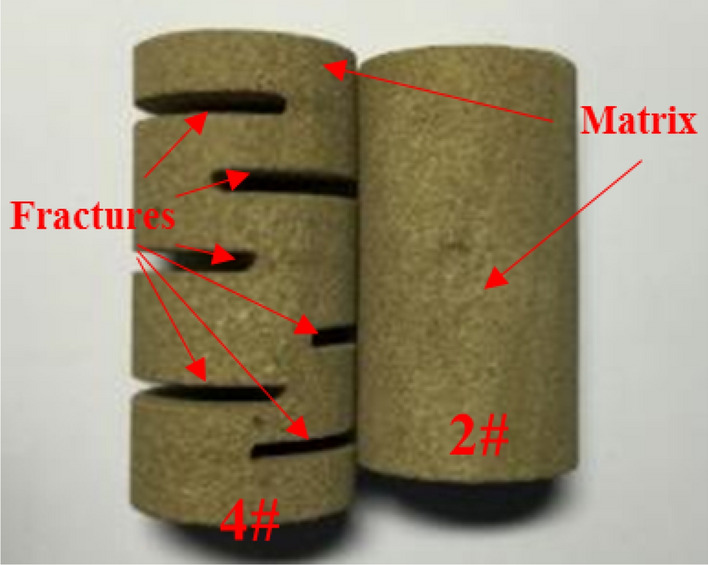
The picture of samples.

The crude oil and cores were collected from CHANG 8 layer of Ordos Basin in Honghe Oilfield with the reservoir pressure and temperature of 12 MPa and 40 °C. The Density and viscosity of oil were determined to be 0.819 g/cm^3^ (at 20 °C) and 5.5 mPa.s (at 40 °C), respectively, at atmospheric pressure. Minimum miscibility pressure (MMP) determined by slim tube test at 40 °C is 17.8 MPa, which is higher than current reservoir pressure. The procedures to clean the core are described as follows. First, the core was cleaned with toluene for two weeks and ethanol for one week. Second, the core was dried in an oven for two days at 105 °C. Then a NMR T_2_ test of cleaned core was performed. The results showed that there was no signal of fluid in these cleaned cores. The core plugs were performed for helium porosity and N_2_ permeability. Their porosity and permeability are shown in Table 1. After the measurement of porosity and permeability, sample 4# was cut to the half of the cylinder six times to increase the external surface area in order to simulate the fracturing process (Fig. 4). There are simple fractures model (only external surface area) in the right of Fig. 4 and complex fractures model with vertical fractures (cutting fractures) and simple fractures (external surface area) in the left of Fig. 4. The fractures were not filled by the sands. The exposure experiment device was unconfined, so the shape of fractures didn’t change during CO_2_ exposure experiment. The exposure external surface area of the tight sandstone core with fractures, i.e. ratio of the external surface area and volume, increased to 1.75 times as compared with that without fractures (Table 1).

### Apparatus

Exposure experiments between CO_2_ and matrix were conducted using the previous apparatuses^8^. The key units were NMR measurement system and exposure experiment device compatible for NMR test with a maximum temperature of 80 °C and a maximum pressure of 35 MPa. A fluorocarbon based heat transfer fluid was used to keep constant temperature in oil bath circulating system. Exposure experiment device was horizontally placed inside NMR. Core with outer diameter of 2.5 cm and two teflon plugs with grooves on the surface were placed in the unconfined cell with inside diameter of 2.7 cm of exposure experiment device and freely contacted the bottom of cell. Two teflon plugs were used to fix the location of core. Injected CO_2_ could flow axially and radially and contact core by grooves on the surface of teflon quickly. When CO_2_ flowed into collection cylinder from exposure experiment device, the collecting syringe pump remained constant pressure of 12 MPa and went backward.

The NMR measurement system (PKU-NMR, Peking University, China) had a permanent magnets (2350 Gauss with an error ± 100 Gauss). Its resonance frequency is 10 MHz. Measurement parameters are shown as follow: Echo spacing: 0.23 ms; Waiting time: 2 s; Echo numbers: 4096; Numbers of scan: 64. After the measurements, transverse relaxation time (T_2_) were calculated by multi-exponential inversion of the echo data with 64 preset decay times logarithmically spaced from 0.1 ms to 10 s. The saturation precision of the NMR being used was 0.5 percent. Laplace transformation in the inversion was used to get the T_2_ relaxation time spectrum and the number was between 30 and 128. The smoothing factor was 0.3.

### Experimental procedure

The experimental procedures and conditions used in this study is briefly described as follow: First, the cleaned cores with or without fractures were vacuumed up to 10^−9^ MPa for 48 h using turbo molecular pump. Second, the cleaned cores with or without fractures were saturated with the crude oil of Honghe oilfield at 80 °C and 30 MPa for 14 days. Third, exposure experiment between CO_2_ and a core was stated: a saturated core was enclosed into the exposure experiment device. After the temperature of the whole system achieved the setting experimental temperature of 40 and became stable, CO_2_ injected into the exposure experiment device from inlet until system pressure reached the experimental pressure of 12 MPa. Then NMR T_2_ test of the core was continuously performed using the NMR system until the obtained T_2_ spectrum kept unchanged. Fourth, the next saturated core was performed the CO_2_ exposure experiment according to the former procedure until those of the four cores with and without fractures were completed.

In the procedure of core vacuum and core saturation core would be better saturated under the condition of high vacuum and 80 °C and 30 MPa for 14 days. The procedure of CO_2_ injection and exposure experiment simulated CO_2_ injection and soaking processes, respectively, during CO_2_ fracturing and EOR processes. The oil mobilization of core was detected and determined using NNR T_2_ spectrum.
